# The Suprapubic and Vaginal Methods of Treating Pelvic Suppuration1Read before the Section of Obstetrics, Buffalo Academy of Medicine, January 28, 1902.

**Published:** 1902-04

**Authors:** W. R. Pryor

**Affiliations:** New York


**BUFFALO MEDICAL JOURNAL.**

VOL. XLI.—LVII. APRIL, 1902. NO. 9.

### ORIGINAL COMMUNICATIONS.

### The Suprapubic and Vaginal Methods of Treating Pelvic Suppuration.[1]

By W. R. PRYOR, M. D., New York.

IT SEEMS to me that very much of the discussion upon this subject arises from the "personal equation" of those who operate for this condition; and were it not for the partisan spirit shown by the advocates of one or the other method, we would be much nearer the truth than we are. Every man should operate by that method which will give him the best results. That is the personal; the scientific is apart from that. There are still some problems to be solved and we can all find sufficient glory in their explanation. I shall not take up your time in boasting of my results in vaginal work, nor in drawing a contrast to the detriment of any colleague who prefers to do laparatomy. I shall attempt to lead you into a discussion upon the principles which should govern us.

I will take first the acute cases, those in which the infection has but recently and for the first time passed outside of the uterus to tubal walls, ovary or peritoneum. It is an undoubted fact that the accepted routine and established method of treatment with by far the greater portion of the profession is to let these cases alone while waiting for a balance to be established between the infection and the tissue-resistance. You all recall the arguments pro and con regarding the question whether or not the gonococcus could cause peritonitis. We now know it can, but that it is not particularly irritating to the peritoneum. It is to the membranes lined by glandular or columnar epithelium that it does its damage. We also have begun to believe

1. Read before the Section of Obstetrics, Buffalo Academy of Medicine, January 28, 1902.

that where gonorrheal infection destroys one tube it also infects the other; that is, that it is always bilateral. We also know how prone such an infection is to cause pyosalpinx or at least occlusion of the tube and such disintegration of the tubal walls as to render them useless for any normal function. For instance, I have never heard of an undoubted and well authenticated instance where a woman who has had gonorrheal salpingitis has subsequently conceived and borne a child. The question then is whether there is any possibility of checking this infection once it has passed outside the uterus, or are the tubes doomed? Now it is a significant fact that we can open the posterior cul-de-sac, remove ectopic sacs, ovarian cysts, sever adhesions and drain with gauze, without subsequent pregnancies being prevented or interfered with. Therefore a pelvic drainage is not damaging to the functions of the ovaries and tubes. We have also shown that such incision of the lowest pouch of the peritoneum is devoid of danger. It is equally well understood that the invasion of a locality by pyogenic cocci depends for much of the damage it can do, upon retention of the products of inflammation. In other words, a free escape of the results of the inflammation as fast as they are produced is inimical to the progress of the inflammation, and that the latter does not proceed to that degree which it would reach were the discharges retained. The tissue-resistance and repair are greatest where drainage is best. That I believe is axiomatic.

For many years I have felt the necessity of placing gonorrheal ovaries and tubes under the benefits of these laws. Can we through the abdomen secure such good drainage of acutely inflamed gonorrheal tubes? If we attempt it through the abdomen is there not risk of producing a more general infection of the peritoneum? This conservative work upon inflamed tubes at the very beginning of the infection, for the purpose of limiting the infection and preventing pyosalpinx, I am most enthusiastic about. But I believe it is useless unless tried through the vagina, and worse than illogical if attempted through the abdomen.

Let us leave the instance of gonorrhea and pass to acute sepsis, the other most frequent cause of suppuration in tubes and ovaries. I believe it is proven beyond doubt that this form of infection always follows some trauma inflicted upon the uterus; therefore, a raw surface is necessary for its inception. Why? Because sepsis travels to and produces lesions in remote organs not by direct continuity of lining membranes, but through the medium of the lymphatics. It in this way has a tendency to spare the essential structures of the tubes, and pyosalpinx may be termed an indirect result of this infection. Again the question arises, cannot something be done to arrest sepsis in its course, or is it better to let the patient alone? If drainage is of benefit in the less virulent cases of gonorrhea, does not the experience of all of you tell you that in other parts of the body, the flesh, the joints, the serous pleural cavity, it is of inestimable value and often as a sole agent is curative.

I have instituted certain experiments with the assistance of the pathologists. We have examined the uterine discharges in septic cases, we have opened the peritoneal pouch and from that have collected fluid, and we call septic for the purpose of this discussion only those cases in which streptococci are found in the peritoneal cavity. After subjecting these cases to a certain line of treatment we have removed and examined the dressings.

It is significant that in no instance have we failed to destroy the causative streptococci and in no instance has death ensued where lesions in remote organs have not been present at the time of the operation. Furthermore, very many of these women have conceived and borne children. We have succeeded in checking the sepsis and a restoration to physiological activity has resulted. Such are the results I have found to follow my treatment through the vagina of acute septic conditions. I would ask you whether in cases of acute septic salpingitis and peritonitis it is advisable to open the abdomen for purposes of merely treating the infection?

Possibly you have not asked me here to discuss this phase of the subject, but those cases in which the sacculation of pus has already taken place. Let us further classify these cases and their treatment. We have those in whom we perform a merely evacuative operation and those in whom we seek a restoration to health by the removal of diseased structures.

Many of you will recall the days when we used to feel large pus foci reaching to the abdomen; how we would pass down to them probes or needles seeking to furnish a very small channel through which a few days later we could evacuate the pus extraperitoneally. In no day of the past or present has any surgeon ever succeeded in evacuating through the abdomen such pus pockets unless he did so by rendering the tract extraperitoneal. But how unfortunate the results usually were. Only in the case of broad ligament abscess were the results anything like satisfactory. Furthermore, the entire length of such drainage tracts were infected and all the organs touching them were involved. The vagina does not absorb pus. Pelvic pus is below the pelvic brim, usually completely locked in. To evacuate it through the vagina is to let the abdominal cavity alone; is to secure escape of the pus at the lowest point and through a natural drainage tract which does not become infected by the discharges.

But you will ask me to pass to the question of removal of pus foci. So far as gonorrheal pyosalpinx is involved, I have to again express my belief that it is always bilateral though of differing degree on the two sides. Therefore the indication is to remove the adnexa of both sides. But would you leave the uterus? Gonorrheal endocervicitis has been shown to be one of the most difficult diseases to cure. The uterus robbed of its supports may become displaced; it is forever becoming reinfected and is a constant source of infection to others. Therefore, whenever I remove the gonorrheal adnexa I take away also the uterus. This being my position, the only question is how the operation shall be done. I find it easier for me and safer for my patients to remove a purulent mass through the vagina, than through the abdominal cavity between the abdominal contents and through an abdominal incision which I hope will unite properly.

But in septic pyosalpinx and ovarian abscess the lesions are very often unilateral. To successfully remove a pus tube we must dig it out of the cornu. It will not suffice to tie it close to the cornu any more than to so treat the suppurating vermiform appendix. Can this be done through the vagina? Undoubtedly not. I am therefore an uncompromising advocate of abdominal section for the removal of unilateral pyosalpinx and ovarian abscess. How frequent this is I do not know. Surely not in 10 per cent. of the cases. But in all cases of bilateral septic tubo-ovarian suppuration I ignore the socalled value of the uterus and perform vaginal ablation.

But we have found that these advanced septic cases follow abortion and labor, conditions in which the uterus is large. Large uteri may reach above the pelvic brim and suppuration about these is apt to lead to important abdominal lesions, such as appendicitis. When such complications are found to exist we must undoubtedly approach the case from the abdominal side.

So then, gentlemen, we find that as an operation to prevent suppuration and check septicemia, the vaginal section stands alone.

As an operation of a purely evacuative character, again vaginal section is the choice.

As a procedure seeking the removal of uterus and both tubes the vaginal operation is far superior to abdominal section, except where high abdominal complications are found.

For the removal of one purulent set of adnexa, there is no operation equal to the abdominal, and the vaginal operation is not to be thought of.

These are my views, and they are substantiated by my experience in their application.

But the great question is, why let suppuration occur? Blameworthy indeed is he who nowadays can sit idly by and see an infection running riot among a woman's most precious organs. As blameworthy as he who would permit a fulminating appendicitis to destroy life without an effort to save. Much criticism is heard of him who removes great gross pockets of pus, "thus ruining the woman." Is not the greater measure of this blame to be laid at the feet of him who permits them to form?

Nowhere else in the body would one dare to adopt such a policy of procrastination, and it is time that we seek answers to these two questions: can suppuration be prevented, and how?

To my almost complete satisfaction I have answers for them, and they are sufficiently indicated in what I have said. It rests with some one to discover that agent which will destroy the gonococcus without injuring the pelvic tissues. My use of iodine compounds I find kills the streptococci.

We have passed far beyond the technique of operations and mortality. It is the morbidity we must in future control. The man who first sees these cases is the one we must instruct and appeal to; for upon the first treatment depends the fate of the involved organs.

121 East Thirty-Eighth Street.

